# Pigment Epithelium-Derived Factor Secreted from Retinal Pigment Epithelium Facilitates Apoptotic Cell Death of iPSC

**DOI:** 10.1038/srep02334

**Published:** 2013-08-01

**Authors:** Hoshimi Kanemura, Masahiro J. Go, Naoki Nishishita, Noriko Sakai, Hiroyuki Kamao, Yoji Sato, Masayo Takahashi, Shin Kawamata

**Affiliations:** 1 Foundation for Biomedical Research and Innovation, Kobe, Japan; 2 Laboratory for Retinal Regeneration, RIKEN Center for Developmental Biology, Kobe, Japan; 3 Division of Cellular and Gene Therapy Products, National Institute of Health Sciences, Tokyo, Japan; 4 Department of Ophthalmology, Kawasaki Medical School, Okayama, Japan

## Abstract

We show that pigment epithelium-derived factor (PEDF), which is secreted from primary or iPSC-derived retinal pigment epithelium (RPE), dramatically inhibits the growth of iPSCs. PEDF is detected abundantly in culture supernatants of primary or iPSC-derived RPE. Apoptotic cell death is induced in iPSC when co-cultured with RPE, a process that is significantly blocked by addition of antibody against PEDF. Indeed, addition of recombinant PEDF to the iPSC cell culture induces apoptotic cell death in iPSCs, but the expression of pluripotency related-genes is maintained, suggesting that PEDF causes cell death, not differentiation, of iPSCs. To recapitulate this event *in vivo*, we examined tumor formation in NOG mice after subcutaneous injection of iPSCs with or without an iPSC-derived RPE sheet (2.5 × 10^5^ RPE cells). We observed that the tumor forming potential of iPSCs was significantly suppressed by simultaneous transplantation with an iPSC-derived RPE sheet.

Cell therapy using embryonic stem cells (ESC) or induced pluripotent stem cells (iPSC) has already entered the scope of clinical application. Indeed, a clinical trial using ESC derived-RPE cells for Stargardt's disease and the dry type of age-related macular degeneration (dry AMD) has been initiated^1^. Clinical trials using autologous iPSC-derived RPE for the wet type of age-related macular degeneration (wet AMD) are also being planned by several groups^2^.

However, tumor formation from residual undifferentiated iPSCs or ESCs is an issue to be evaluated carefully in the transplantation of pluripotent stem cell-derived tissue products. This issue becomes more serious in the case of transplanting autologous iPSC-derived cells or tissues at sites lacking an immune barrier. The tumor forming potential of the remaining undifferentiated iPSCs in iPSC derived-cell products should be examined by taking into account the number of iPSC-derived cells to be transplanted, and the micro-environment of the transplantation site. The method and its sensitivity to detect the remaining iPSCs are also key issues to assure the safety of transplantation of iPSC-derived cell products.

We recently reported a method that was highly sensitive for the detection of residual iPSCs in iPSC-derived retinal pigment epithelium (RPE). It relied on qRT-PCR using primers for the *LIN28A* transcript^3^. With this method, we could theoretically detect iPSCs equivalent to 0.01% of the total cell product. Considering the fact that we plan to transplant 4 − 8 × 10^4^ iPSC-derived RPE cells in a clinical setting, we should be able to detect the few residual iPSCs in the iPSC-derived RPE prior to transplant. Apart from the development of a sensitive residual iPSC detection method, it is important to explore the paracrine effects originating from differentiated iPSCs and/or host tissues on residual iPSCs. Secreted factors could have profound effects on iPSCs and their derived products after transplant. For example, RPE is known to secrete a variety of cytokines, connective tissue proteins, extracellular matrix proteins, complement factors, proteases, and protease inhibitors^4^. In this report, we studied the non-autonomous trans-effects of RPE on iPSCs and discuss the safety concerns for tumor formation from residual iPSCs in iPSC-derived RPE.

## Results

### Differentiation of iPSC into RPE cells

**Figure 1:**
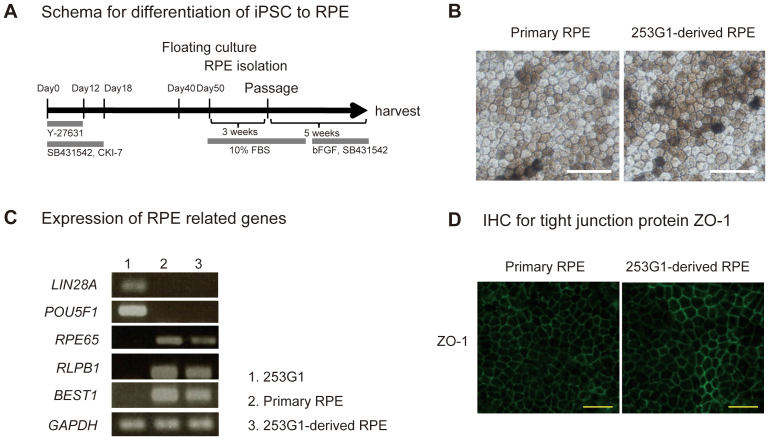
Characterization of pigment epithelial cells derived from iPSC. (A) Protocol for differentiation to RPE from iPSC clone. (B) Phase contrast images of primary RPE (left panel) and iPSC clone 253G1-derived RPE (right panel). Scale bar = 50 μm. (C) Expression of pluripotency-related undifferentiated marker genes (*LIN28A* and *POU5F1*) and RPE-specific genes (*RPE65*, *RLPB1*, and *BEST1*) detected by qRT-PCR. *GAPDH* was used for internal gene expression control. (D) Immunofluorescence staining of tight junction protein ZO-1. Secondary antibody Alexa 488 was used to visualize the staining. Scale bar = 50 μm.

In an effort to establish a robust differentiation protocol for pluripotent stem cells into retinal pigment epithelium (RPE), the differentiation protocol shown in Figure 1A was used. In this report, we used a commercially available iPSC clone 253G1^5^ (Riken Bio Resource Center, Tsukuba Japan) as a cell source for RPE differentiation to present a reproducible profile of iPSC-derived RPE. RPEs are sporadically pigmented, polygonal in shape, and grow in monolayers when cultured in dishes. iPSC clone 253G1 derived-RPE and primary RPE showed the same morphology in microscopic observation (Fig. 1B). To determine whether iPSC-derived RPE cells possessed the characteristic gene expression of primary RPE, the expression of *RPE65*, *RLPB1*, and *BEST1* was analysed by RT-PCR. 253G1-derived RPE cells expressed the *RPE65*, *RLPB1*, and *BEST1* messages, but not pluripotency-related genes such as *LIN28A* and *POU5F1* (Fig. 1C). Tight junction specific protein, ZO-1 was also detected both in 253G1-derived RPE and primary RPE by immunofluorescent staining (Fig. 1D).

### Cell growth of iPS cells co-cultured with iPSC-derived RPE was drastically perturbed

**Figure 2:**
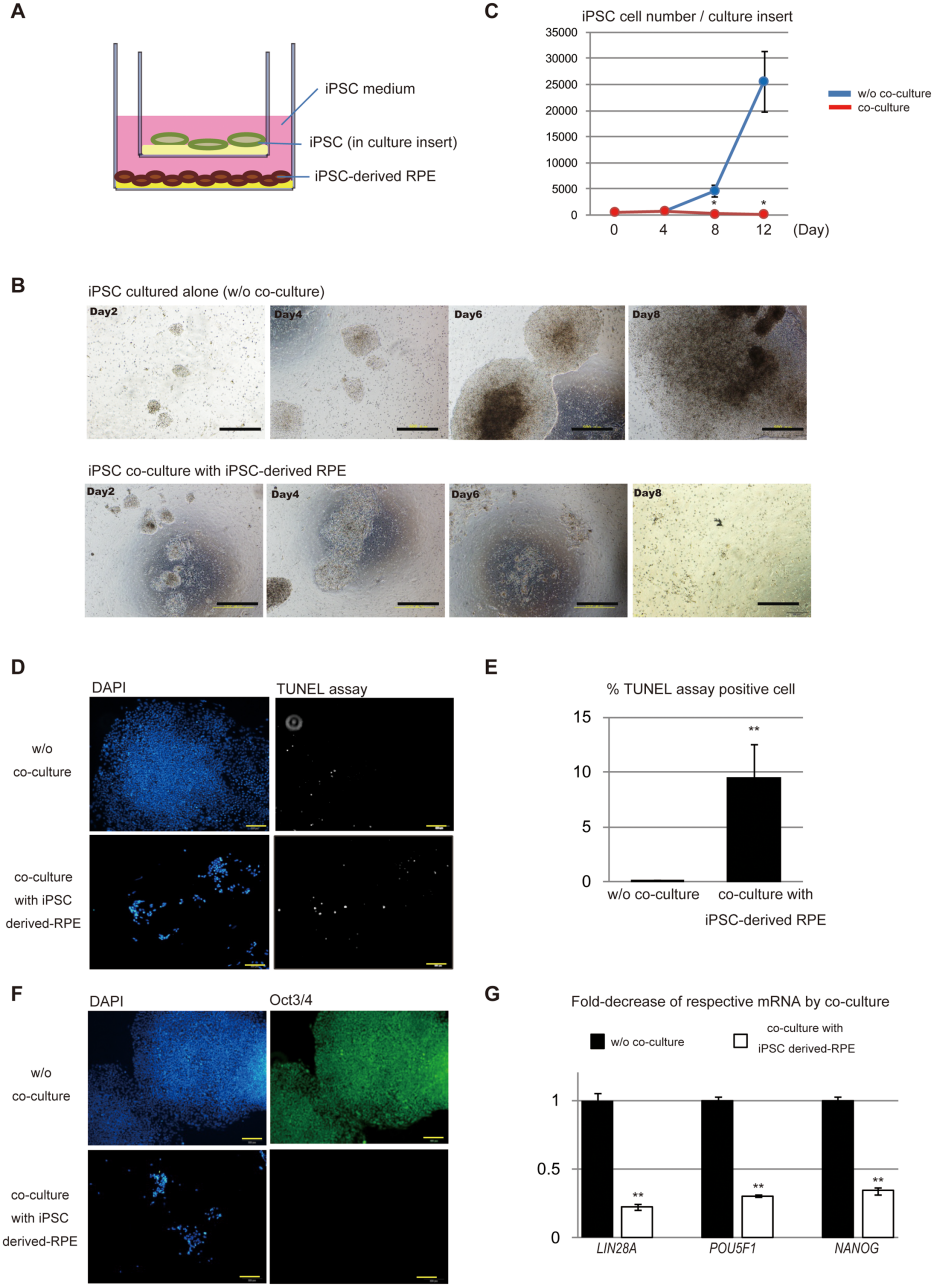
Cell growth of iPSCs co-cultured with iPSC-derived RPE was perturbed. (A) Schema for co-culturing iPSC with RPE. iPSCs were maintained in culture inserts coated with Matrigel and co-cultured with iPSC-derived RPE seeded on the bottom of the dishes in iPSC culture medium. (B) Phase contrast photos of iPSC clone 253G1 in 12-well Transwells either cultured alone or co-cultured with 253G1-derived RPE on the designated day of culture. Scale bar = 500 μm. (C) Growth curve of iPSC clone 253G1 co-cultured with 253G1-derived RPE or cultured alone (w/o co-culture). The number of iPSC clone 253G1 cells in 12-well Transwells at the designated day of culture was scored. Means of three independent experiments are plotted on a linear graph with standard deviation (SD). *, *P* < 0.05, compared as indicated. (D) Apoptotic cell death was examined (or analyzed) by TUNEL assays and visualized as white spots on day six of culture. Scale bar = 200 μm. (E) Ratio of TUNEL-positive cells to DAPI positive 253G1 cells (as a percentage) either cultured alone (w/o co-culture) or co-cultured with 253G1-derived RPE. Mean results (with SD) from four independent experiments. **, *P* < 0.005, compared as indicated. (F) 253G1 cells co-cultured with 253G1-derived RPE markedly lost the expression of undifferentiated marker Oct3/4 (POU5F1) after six days of culture. Cells were stained with antibody for Oct3/4 (POU5F1), and then visualized with secondary antibody Alexa 488 (green, right panels). Nuclei were stained with DAPI (blue, left panels). Scale bar = 200 μm. (G) Fold-decrease of indicated mRNAs in iPSC resulting from co-culturing with iPSC-derived RPE. mRNA levels of *LIN28A, POU5F1* or *NANOG* in 235G1 were measured by quantitative RT-PCR. *GAPDH* was used as an internal control to normalize the mRNA levels of these genes. Mean results (with SD) derived from three independent experiments. **, *P* < 0.005, compared as indicated.

To explore the effect of factors secreted by iPSC-derived RPE on iPSCs *in vitro*, we conducted co-culture experiments (Fig. 2A). iPSCs seeded on Matrigel-coated culture (Transwell) inserts were co-cultured with iPSC-derived RPE seeded on CELL start-coated dishes in iPS medium (ReproFF2 supplemented with bFGF). The iPSCs in the culture insert were harvested every four days and the cell number was scored. We found that the proliferation of iPSC was significantly inhibited by co-culturing with iPSC-derived RPE (Fig. 2B,C). It is notable that a similar trans-effect was observed when iPSCs were co-cultured with primary RPE (Supplementary Fig. 1A–C). Marked inhibition of the growth of iPSCs co-cultured with iPSC-derived RPE was, at least partly, mediated by apoptotic cell death, as shown by the presence of TUNEL-positive cells (Fig. 2D, E). Furthermore, immunostaining and qRT-PCR study of the remaining iPSCs in co-culture showed that the expression of pluripotent-related genes (such as *LIN28A*, *POU5F1*, and *NANOG*) was markedly reduced, suggesting that the conditioned medium from iPSC-derived RPE induced cell death and at the same time promoted differentiation of iPSCs (Fig. 2F,G).

This observation prompted us to explore the factors from iPSC-derived RPE and primary RPE that had a trans-effect on iPSC cell proliferation. We performed microarray analysis with the GeneChip® system (Affymetrix), studying primary RPE, the 253G1-derived RPE and the parent iPSC clone 253G1. Several secreted factors were identified, with high message expression in both primary RPE and iPSC-derived RPE but only low/no expression in iPSC. For example, pigment epithelium-derived factor (PEDF), vascular endothelium growth factor (VEGF), bone morphogenetic protein 4 (BMP4), microsomal glutathione S-transferase (MGST), and glutathione S-transferase mu3 (GSTM3) showed high levels of message [Supplementary Table 1 and Data Set in GEO http://www.ncbi.nlm.nih.gov/gds (GEO number: GSE43257)]. Among those molecules, PEDF, VEGF, BMP4 have been reported to affect differentiation, proliferation, migration, and apoptosis^9^,^10^,^11^ Thus, they were extracted and examined for a trans-effect on iPSCs.

### Apoptotic cell death of iPSC is partly mediated by PEDF

**Figure 3:**
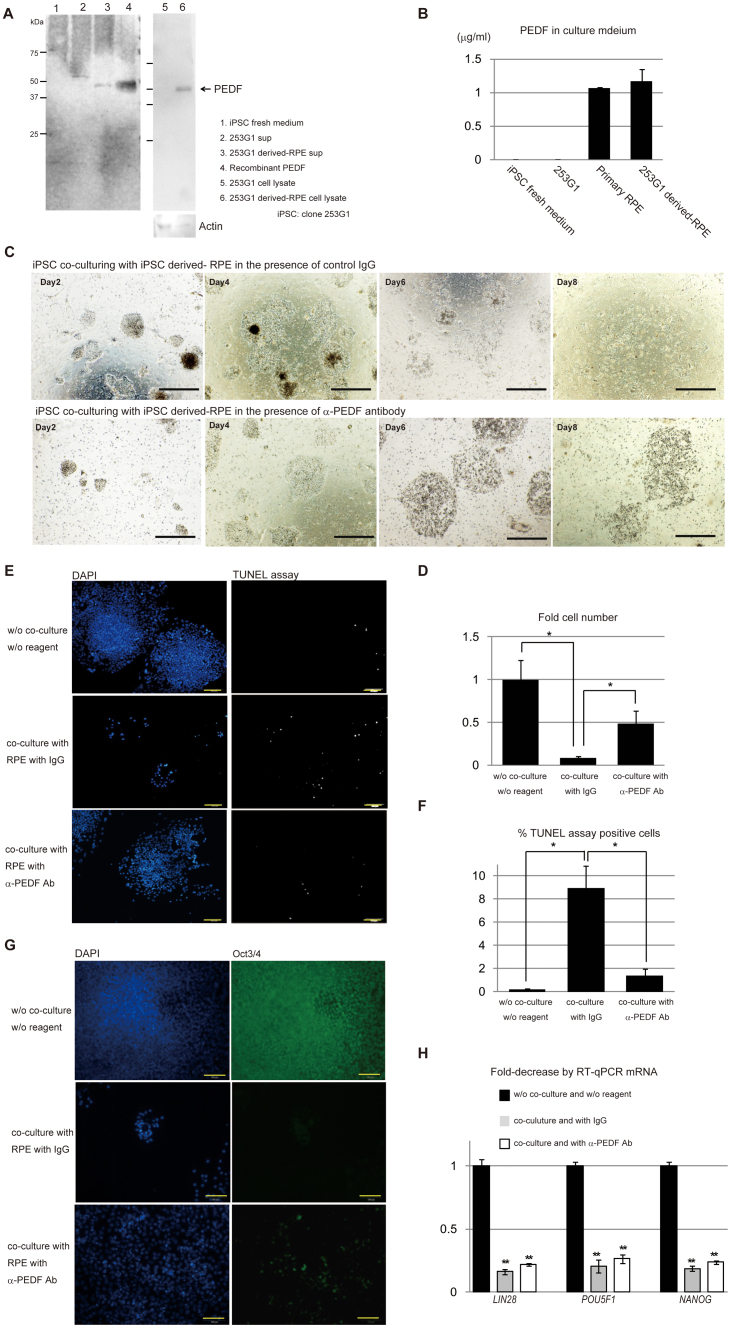
Addition of antibody against PEDF blocked apoptotic cell death in iPSC induced by co-culture with RPE. (A) Western blot of iPS fresh culture medium, 253G1 sup, 253G1-derived RPE sup and recombinant PEDF, or cell lysates of 253G1 and 253G1-derived RPE using an anti-PEDF specific antibody. Detection of actin was used as a loading control for cell lysates. (B) Quantitation of PEDF in iPSC fresh culture medium, 253G1 sup, primary RPE or 253G1-derived RPE conditioned medium by ELISA. Mean results of three independent experiments (with SD). (C) Phase contrast images of 235G1 co-cultured with 253G1-derived RPE in the presence of control IgG1 or anti-PEDF antibody on the designated day of culture. Scale bar = 500 μm. (D) Fold-change in the number of 253G1 cells co-cultured with 253G1-derived RPE in the presence of IgG1 or anti-PEDF antibody after six days of incubation. Cell counts were compared to 253G1 cultured alone without reagent. Mean results of four independent experiments (with SD). *, *P* < 0.05. (E) 235G1 co-cultured with 253G1-derived RPE in the presence of control IgG1 or anti-PEDF antibody after six days of culture were examined by TUNEL assay visualized as white spots. 253G1 cultured alone without reagent was used as the control. Scale bar = 200 μm. (F) Ratio of TUNEL assay-positive 253G1 cells to DAPI positive cells when 253G1 cells were co-cultured with 253G1-derived RPE in the presence of control IgG1 or anti-PEDF antibody after six days of incubation. Mean results (with SD) from four independent experiments. *, *P* < 0.05, compared as indicated. (G) 253G1 cells co-cultured with 253G1-derived RPE markedly lost the expression of undifferentiated marker Oct3/4 (POU5F1) after six days of incubation. Cells were stained with antibody for Oct3/4, and then visualized with secondary antibody Alexa 488. Nuclei were stained with DAPI. Scale bar = 200 μm. (H) Fold-decrease of indicated mRNAs in iPSC resulting from co-culturing with 253G1-derived RPE. mRNA levels of *LIN28A, POU5F1* and *NANOG* were measured by quantitative RT-PCR. *GAPDH* was used as an internal control to normalize the mRNA expression levels. Mean results of three independent experiments (with SD). **, *P* < 0.005.

Using a specific anti-PEDF antibody, PEDF protein (with a size of 50 kDa) was detected by Western blotting (Fig. 3A) in the conditioned medium of iPSC-derived RPE, and in cell lysates of both iPSC and iPSC-derived RPE. Fresh iPSC medium (medium without co-culturing) was used as a control sample. The amount of PEDF present after 24 h of cell culture (24 hours after changing with fresh medium) was measured by ELISA. The conditioned media from both primary RPE and iPSC-derived RPE contained a considerable amount of PEDF (more than 1 μg/mL) (Figure 3B).

VEGF and BMP4 in the conditioned media from primary RPE or iPSC-derived RPE cell cultures were detected by ELISA (Supplementary Fig. 2A,B). However, addition of 0.1 μg/mL, 5 μg/mL, or 20 μg/mL of recombinant VEGF (rVEGF, Peprotech), or 0.02 μg/mL, 1 μg/mL, or 4 μg/mL of rBMP4 (Peprotech) failed to alter cell growth markedly (Supplementary Fig. 2C). Then, we examined the effect of PEDF on the growth of iPSCs. To address this, a specific neutralizing antibody for PEDF (BioProducts, MD) was added to the co-culturing system and the proliferation of iPSCs in the culture inserts was examined. Growth inhibition of 253G1 cells co-cultured with 253G1-derived RPE was observed in the presence of control IgG. However, growth inhibition was efficiently blocked by anti-PEDF antibody (Fig. 3C). Almost half of the iPSCs were rescued by addition of 5 μg/mL polyclonal anti-PEDF neutralizing antibody (Fig. 3D). Specifically, it appeared that neutralizing antibody against PEDF reduced apoptotic death of iPSCs (Fig. 3E, 3F). Based on this experiment, we concluded that PEDF induced cell death of iPSCs.

Next, we examined whether PEDF could promote differentiation of iPSCs as well as induce cell death. iPSCs co-cultured with RPE in the presence of control IgG initiated differentiation as evidenced by a decrease of *LIN28A, POU5F1* and *NANOG* message levels. This message reduction was not attenuated by the addition of anti-PEDF antibody (Fig. 3G, 3H), suggesting that PEDF contributed to the induction of iPSC death but not to iPSC differentiation. VEGF and BMP4, known to induce pluripotent stem cell differentiation, were also detected in the RPE-conditioned medium by ELISA (Supplementary Fig. 2 A, B). We hypothesize that those factors could contribute to the differentiation of iPSCs. However, most iPSCs are subject to cell death by PEDF in RPE-conditioned medium (Fig. 2B, C). Thus, the differentiation of the remaining iPSCs induced by these factors, if any, might well be masked.

To directly address the effects of PEDF on the growth of iPSC, we used recombinant PEDF protein (rPEDF, Millipore). The biological activity of procured rPEDF was titered with human umbilical vein endothelial cells (HUVEC), as PEDF reportedly has anti-angiogenic function^12^. Indeed, the conditioned medium from RPE showed a cell growth inhibitory effect on HUVEC (Supplementary Fig. 3A). Thus, we examined several doses of rPEDF (Supplementary Fig. 3B) for its growth inhibitory effect on HUVEC. We found that 50 μg/mL PEDF possessed a biological effect on HUVEC comparable to that of 1/4 volume of conditioned medium mixed with HUVEC medium (M-200 supplemented with LSGS). There was no cell growth inhibitory effect under 50 μg/mL of rPEDF. Therefore, we used 50 μg/mL of rPEDF for further examination of the effect of rPEDF. At 50 μg/mL rPEDF, we observed increased apoptosis in HUVECs (Supplementary Fig 3C), as well as a growth inhibitory effect (Supplementary Fig 3D). To rule out the possibility that the high dose of recombinant protein contained various non-specific factors that might have non-specifically induced cell death, neuroblastoma SK-N-BE (2) and primary RPE cells were cultured with 50 μg/mL of rPEDF. We found that 50 μg/mL rPEDF did not change either the morphology or reduce the number of neuroblastoma cells (Supplementary Fig. 3E) or primary RPE (Supplementary Fig. 3F).

**Figure 4:**
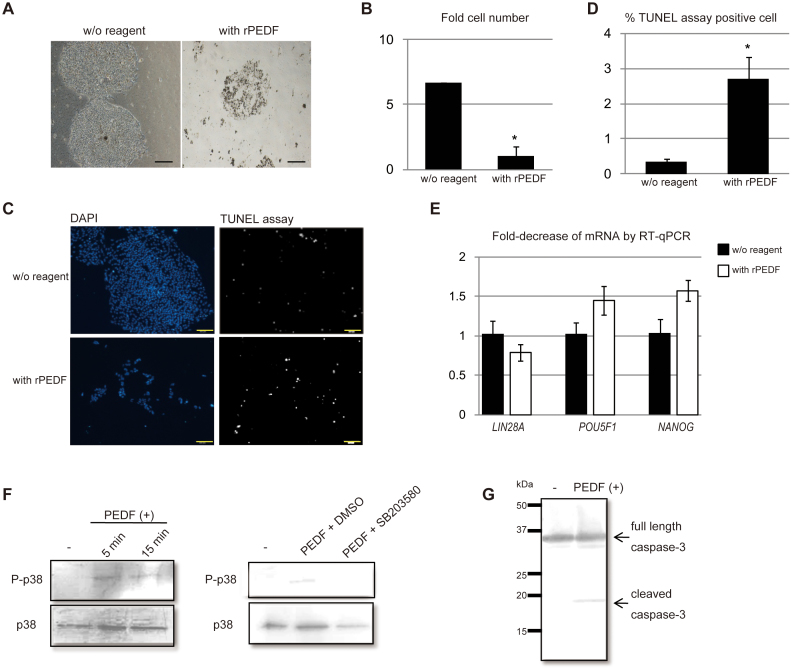
Recombinant PEDF (rPEDF) induced apoptotic death in iPSCs. (A) Phase-contrast images of iPSC clone 253G1 without or with rPEDF (50 μg/mL) after four days of culture. Scale bar = 200 μm. (B) Fold-change in the number of 253G1 cells cultured with rPEDF (50 μg/mL) after four days of culture, compared with the number of 253G1 cells cultured without rPEDF. Mean results of three independent experiments (with SD). *, *P* < 0.05, compared as indicated. (C) Apoptotic death of 253G1 cells was examined by the TUNEL assay and visualized as white spots (right) after four days of culture. Nuclear staining with DAPI is shown on the left. Scale bar = 200 μm. (D) Ratio of TUNEL positive cells to DAPI positive cells, as a percentage, when cultured with or without recombinant PEDF. Mean results of three independent experiments (with SD). *, *P* < 0.05, compared as indicated. (E) mRNA levels of *LIN28A, POU5F1* and *NANOG* in 253G1 cells after four days of culture without or with rPEDF (50 μg/mL) were measured by qRT-PCR. *GAPDH* was used as an internal control to normalize mRNA expression levels. Fold-decrease or increase of respective mRNAs in iPSC. Mean results of three independent experiments (with SD). (F) Left panels: phosphorylated p38 MAPK (P-p38) or p38 MAPK (p38) after six hr serum starvation of iPSCs (−), and five min (5 min) or 15 min (15 min) after addition of PEDF (50 μg/mL) [PEDF(+)]. Proteins were detected by Western blotting with specific antibody. p38 was used as an internal control. Right panels: phosphorylated p38 MAPK (P-p38) in serum starved iPSCs (-), 10 min after addition of PEDF (50 μg/mL) in the absence (PEDF + DMSO) or presence of p38 inhibitor SB203580 (PEDF + SB203580). Proteins were detected by Western blotting. (G) Cleaved Caspase-3 after six hr serum starvation of iPSCs [−], or ten min after addition of PEDF (50 μg/mL) [PEDF(+)] was detected by Western blotting with specific antibody.

One plausible explanation for the marked gap in dosage between the amount of PEDF in the conditioned medium and the biologically relevant dose of rPEDF would be low biological activity of rPEDF due to altered post-transcriptional modification of PEDF when it is produced in Baby Hamster Kidney cells. Fifty μg/mL rPEDF inhibited cell growth of iPSC (Fig. 4A,B) and induced apoptotic cell death as evidenced by TUNEL assay (Fig. 4C,D). It is interesting to note that 50 μg/mL of rPEDF also induced apoptotic cell death in human ES cells (khES01) (Supplementary Fig.4). The morphology of the remaining iPSCs after rPEDF addition was the same as untreated iPSC (Fig. 4A). Moreover, reductions in the messages of pluripotency related-genes *LIN28A*, *POU5F1*, and *NANOG* in the remaining cells were not observed (Fig. 4E). The cell number counted by scoring DAPI-positive cells after rPEDF treatment was not constant. That may account for the up-regulation of message of pluripotency related-genes after addition of rPEDF.

We next explored the PEDF-mediated signal pathway leading to apoptosis in iPSCs. Western blotting detected phosphorylation of p38 mitogen-activated protein kinase (MAPK) and cleaved caspase-3 after rPEDF stimulation of iPSC (Fig. 4F,G). Taken together, it is conceivable that PEDF induced the apoptotic death of iPSC, but did not induce differentiation of iPSC.

### RPE cell sheet suppressed tumor formation potential of iPSC when co-transplanted *in vivo*

**Figure 5:**
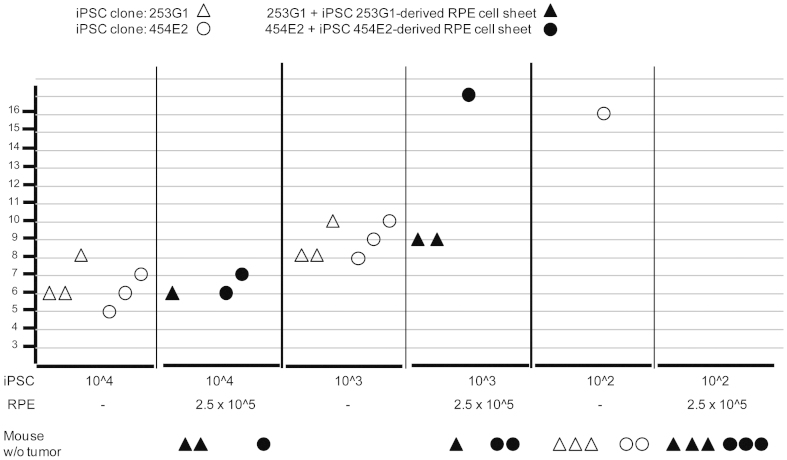
iPSC clones 253G1 or 454E2 (10^2^, 10^3^ and 10^4^ cells) were subcutaneously transplanted to NOG mice (three per group) without or with a 253G1-derived RPE sheet or a 454E2-derived RPE sheet, respectively. The sheets consisted of (approximately) 2.5 × 10^5^ RPE cells. The Y-axis shows the week when the tumors were first detected in each case. The number of iPSC cells and iPSC-derived RPE are shown on the X-axis. The numbers of mice without tumor formation are shown as symbols below the X-axis.

We plan to transplant a cell sheet of RPE to the retinas of patients who suffer from aged macular degeneration. Specifically, we will use one to two RPE cell sheets (1.3 mm × 3 mm), consisting of approximately 2 − 5 × 10^4^ RPE cells. The RPE sheet is prepared on a collagen gel [Kamao H, et al. manuscript submitted]. The possibility of tumor formation from residual undifferentiated iPSCs or incompletely differentiated cells in an iPSC-derived product after transplant remains an issue. To evaluate the trans-effect of RPE on the remaining iPSCs after transplant to the retina, we set up a series of iPSC “spike tests” in the presence of RPE sheets using immunosuppressed animals. We tested the tumor formation potential in several immunosuppressed animals by injecting several doses of iPSCs either subcutaneously or in the retina. Recipient animals included rat (nude rat: F344/NJcl-rnu/rnu) and mouse (Nude: BALB/cA, JCl-nu/nu; SCID: C.B-17/Icr-scid/scid, Jcl; NOD-SCID: NOD/ShiJic-scid, Jcl; NOG: NOD/ShiJic-scid, IL-2Rγ KO Jic). We found the NOG mouse was the most sensitive animal in terms of tumor formation from iPSCs and HeLa cells when injected subcutaneously with Matrigel (BD), in agreement with a previous report^13^. Then, 10^2^, 10^3^, or 10^4^ iPSCs (clone 253G1 or 454E2) were co-transplanted into NOG mice subcutaneously without or with iPSC clone 253G1 or a 454E2 derived-RPE sheet consisting of approximately 2.5 × 10^5^ RPE cells. The mice were monitored for tumor development at the site of injection for 30 weeks. Three-way ANOVA (factors: dose of iPSCs, clone of iPSCs, presence of RPE cell sheet) and the post-hoc Student-Neuman-Keuls test for latency of tumor formation indicated that tumors appeared significantly earlier in the groups inoculated with 10^3^ or 10^4^ iPSCs, compared with that inoculated with 10^2^ iPSCs. (Fig. 5, *P* < 0.001). More importantly, the statistical analysis indicated a significant difference between the groups with and without RPE sheet (*P* < 0.01), whereas there was no difference between the iPSC clones.

## Discussion

In this report, we demonstrated that both primary and iPSC-derived RPE secreted PEDF that induced apoptosis in iPSC.

To elucidate the mechanism by which the tumor (teratoma) forming capacity of iPSCs was suppressed when the RPE cell sheet was co-transplanted, additional studies are required. It is possible that nonspecific effects of the transplanted RPE sheet could compete with the tumor for endogenous growth substrates. Alternatively, it might induce the host immune system to attack co-transplanted cells or reduce the size of the tumor through an anti-angiogenic effect of PEDF as reported^16^,^17^. However, we have some suggestive data pertinent to this issue. Effect of PEDF on cell growth *in vitro* varies depending on cell type. Indeed, reduction of HeLa cell number was not drastic compared with that of iPSC cell number after the RPE conditioned medium treatment (Supplementary Fig. 5A). In this context, HeLa cells formed tumors when as few as one hundred cells were injected into the retinas of nude rats (TPD_50_ = 32). In contrast, injection of as many as one thousand hiPSCs into nude rat retinas did not generate teratomas (TPD_50_ = 31623) (Supplementary Fig. 5B). These experiments suggested that a non-autonomous effect of RPE *in vivo*, if any, is cell-type specific, and that RPE selectively suppress the growth of iPSCs when iPSCs are transplanted in RPE or co-transplanted with RPE.

Considering the fact that we plan to transplant 4 − 8 × 10^4^ iPSC-derived RPE cells in a clinical setting and have developed a highly sensitive iPSCs detection system using qRT-PCR^3^ (theoretically capable of detecting iPSCs in RPE cells when iPSCs constitute only 0.01% of the total cell product), the chances of a tumor formation from the undetectably low number of residual iPSCs in iPSC-derived RPE cell sheet following the transplantation should be extremely low in the presence of PEDF secreted from RPE.

PEDF is a 50 kDa secreted protein that is also known as serpin F1^14^,^15^. PEDF is reported to possess various biological functions including inhibition of endothelial proliferation^9^,^11^ and angiogenesis^16^,^17^, as well as neurophilic functions^18^,^19^,^20^ and induction of apoptosis^21^,^22^, after binding to its receptor^23^. PEDF enhances gamma secretase activities leading to cleavage of VEGF receptor-1^24^ and VEGF receptor-2^25^, and induces an anti-angiogenic protein, thrombospondin^26^. With regard to angiogenesis and endothelial cell proliferation, RPE secretes both counter-acting PEDF and VEGF. RPE, however, maintains the microenvironment and the structure of the retina by secreting these factors into a different side of the retinal membrane. These facts necessitate RPE sheet transplantation in the proper orientation rather than as single RPE cells to ensure the function of retina. PEDF is reported to promote the differentiation of primitive retinal cells^27^and retinoblastoma cells^19^, but the effect of PEDF on iPSC seems to be limited to induction of apoptotic cell death, not neural differentiation of iPSC.

PEDF reportedly stimulates several signal pathways including activation of Ras, NF-kB^18^, FAS/FASL^12^, PPAR-gamma, and the p53-mediated pathway^21^. The p38 MAPK-mediated cleavage of caspases is also reported in endothelial cells^22^. In this study, we showed the activation of p38 and cleavage of caspase-3 after rPEDF stimulation in iPSC (Fig. 4F). Therefore, it is conceivable that p38 MAPK-dependent cleavage of multiple caspases could lead to apoptosis in iPSC after PEDF stimulation. Recently, it was reported that PEDF activated ERK1/2 and maintained growth of hESC^28^. They used 100 ng/mL rPEDF to show activation of ERK1/2 in serum-starved hESC, and ERK1/2 inhibitor PD98059 inhibited growth of hESC. Based on these experiments, they concluded that PEDF maintained cell growth of hESC via ERK1/2 activation. ERK1/2, key signal molecules, activate multiple signals leading to various biological responses. Blocking ERK1/2 activities will inevitably suppress multiple critical cellular responses and not necessarily address a PEDF specific-signal event. In our experiments, we did not observe a biological process resulting from rPEDF stimulation below 50 μg/mL (Supplementary Fig. 3B). Thus, we believe 100 ng/mL rPEDF might be enough to initiate ERK1/2 signaling, but not enough to initiate cellular events in hiPSC or hESC.

In summary, we showed a novel effect of PEDF on the survival of remaining iPSCs in iPSC-derived RPE and suggest further application of PEDF in pluripotent stem cell-based cell therapy in the future.

## Methods

All the experiments using human samples and animal studies were approved by the IRB of the Foundation for Biomedical Research and Innovation (FBRI) and Riken Center for Developmental Biology (Riken CDB), and the committee for animal experiments of the FBRI.

### Cell culture

Human primary retinal pigment epithelium (RPE, Lonza) was maintained in Retinal Pigment Epithelial Cell Basal Medium (Lonza Biologics, Basel, Switzerland) containing supplements (L-glutamine, GA-1000, and bFGF; Lonza). Human iPS cell (iPSC) lines 253G1^5^ [Riken Bio Resource Center (Tsukuba, Japan)] and 454E2^6^ were maintained on feeder cell SNL^7^ in human ES cell culture medium and 5 ng/mL bFGF (Peprotech). iPSCs were cultured in ReproFF2 (ReproCELL) supplemented with 5 ng/mL bFGF medium. iPSC-derived RPE^3^,^8^ was maintained in RPE maintenance medium [DMEM:F12 (7:3) (Sigma-Aldrich) containing B-27 supplement (Invitrogen), 2 mM L-glutamine (Invitrogen), 0.5 mM SB431542 (Sigma-Aldrich) and 10 ng/mL bFGF (Wako)]. HUVECs (BD™) were maintained in M-200 supplemented with LSGS and neuroblastoma cells (SK-N-BE (2), ATCC) were cultured in DMEM containing 10% FBS.

### Cell growth of iPSCs, HUVECs and neuroblastoma cells in the absence or presence of recombinant PEDF or anti-PEDF antibody

253G1 cells were seeded in Matrigel (BD Bioscience)-coated 12-well Transwell cell culture inserts with an 8 μm pore size (BD). They were co-cultured with primary RPE or 253G1 derived-RPE seeded on the bottom of dishes in ReproFF2 medium supplemented with bFGF in the absence or presence of one to 50 μg/mL rPEDF (Millipore, cat # GF134 lot: DAM 1821182) or 5 μg/mL polyclonal anti-PEDF blocking antibody (cat # AB-PEDF1, BioProducts MD)^29^,^30^,^31^,^32^ or 5 μg/mL non-functional control rabbit IgG (Santa Cruz). Cell growth of 253G1, HUVEC, neuroblastoma and primary RPE in the absence and presence of 50 μg/mL rPEDF was evaluated after four to 6 days of culture (without co-culture).

### Chip analysis

Total RNA from 253G1 or 253G1-derived RPE was isolated with a RNeasy Plus Mini Kit (Qiagen) in accordance with the manufacturer's instruction and hybridized with Gene Chip Human Genome U133 Plus ver. 2.0 (Affymetrix). Hybridized microarray data were scanned with a GeneChip Scanner 3000 7 G. Analyzed data can be retrieved from the GEO http://www.ncbi.nlm.nih.gov/gds/. Our GEO data set number is GSE43257.

### ELISA

Levels of PEDF, VEGF or BMP4 in primary RPE or iPSC (253G1)-derived RPE culture medium (conditioned medium) collected after 24 h of culture were determined with human ELISA kits (PEDF, BioVendor; VEGF, eBioscience; BMP4, RayBiotech) in accordance with the manufacturers' instructions.

### qRT-PCR

Total RNA was isolated with the RNeasy plus Mini Kit (Qiagen) in accordance with the manufacturer's instructions. Contaminating genomic DNA was removed using a gDNA Eliminator spin column. cDNA was generated from one μg of total RNA using PrimeScript RT Master Mix (Takara Bio) and PrimeSTAR MAX DNA Polymerase (TaKaRa Bio). Real-time PCR was then performed with an ABI 7000 Sequence Detection System (Applied-Biosystems) and SYBR-green in accordance with the manufacturer's instruction. The primers designed for real-time PCR were as follows: for *LIN28A*, forward primer, 5'- CTGTCCAAATGCAAGTGAGG-3', reverse primer, 5'-GCAGGTTGTAGGGTGATTCC-3'; for *POU5F1*, forward primer, 5′- GAAGGTATTCAGCCAAACGAC-3', reverse primer, 5′- GTTACAGAACCACACTCGGA-3'; for *NANOG*, forward primer, 5′- CTCAGCTACAAACAGGTGAAGAC-3′, reverse primer, 5′- TCCCTGGTGGTAGGAAGAGTAAA-3′; for *RPE65*, forward primer, 5′- ATGGACTTGGCTTGAATCACTT-3', reverse primer, 5′-GAACAGTCCATGAAAGGTGACA-3′; for *BEST1*, forward primer, 5′-ATCAGAGGCCAGGCTACTACAG-3′, reverse primer, 5′-TCCACAGTTTTCCTCCTCACTT-3′; for *RLPB1*, forward primer, 5′-GACTGGGGTTAAATCTCACAGC-3′, reverse primer, 5′-TGACATGTTGCCTATGGAAGAC-3′; for *GAPDH*, forward primer, 5′- CGATGCTGGCGCTGAGTAC-3′, reverse primer, 5′-CCACCACTGACACGTTGGC3′. Respective gene expression levels were normalized to that of GAPDH*.*

### TUNEL staining and Immunohistochemistry

Apoptotic cells were detected with the *In situ* Cell Death detection kit (fluorescein, Roche Diagnostics) in accordance with the manufacturer's instructions. The percentage of TUNEL-positive cells was calculated by scoring TUNEL-positive cells divided by total DAPI-positive cells in three non-overlapping areas (two mm^2^ per well).

For immunochemical staining, cells were fixed with 4% paraformaldehyde followed by staining with antibodies against Oct3/4 (POU5F1) (1:100 dilution; sc-5279; Santa Cruz), or ZO-1 (1:200 dilution; Invitrogen). Antibodies were visualized with Alexa Fluor 488 goat anti-mouse (1:1,000; Invitrogen) or Alexa Flour 488 goat anti-rabbit (1:1,000; Invitrogen). Fluorescent microscopic images were captured with a fluorescent microscope (Olympus BX51, IX71; Tokyo, Japan).

### Western blotting

Cell culture supernatants (conditioned media) or recombinant protein samples were loaded onto a 5 – 20% gradient SDS-polyacrylamide gel, subjected to electrophoresis under reducing conditions and blotted onto a PVDF membrane (BioRad). Blots were blocked with a solution of 3% nonfat dry milk/PBS/0.1% Tween-20 at room temperature, rinsed twice with PBS/0.1% Tween-20 and incubated with 1:200 diluted polyclonal anti-PEDF antibody (BioProducts MD), followed by 1:5000 diluted anti-rabbit IgG-HRP (Amersham). Detection of actin by anti-actin antibody (Santa Cruz I–19) was used as a loading control. Membranes were rinsed three times in PBS/0.1% Tween-20. Signals were detected with horseradish peroxidase using an ECL kit (Promega). Cell lysates were made from iPSCs that were serum-starved for six h (−) or five min or 15 min after addition of PEDF in the absence or presence of p38 MAPK inhibitor SB203580 (Cell Signaling). Lysates were blotted onto PVDF membranes, and anti-phospho-p38 antibody (Cell Signaling), anti-p38 antibody (Cell Signaling) or anti-caspase 3 antibody (Cell Signaling) was used to detect the respective molecules.

## Author Contributions

H. Kanemura conducted all of the biological assays and prepared the manuscript; M.G. designed the research; N.N. analyzed the gene chip data; N.S. supplied the iPSC and iPSC-derived RPE; H. Kamao supplied the RPE sheets; Y.S. performed the statistical analysis of transplantation experiments; M.T. performed the QC for the RPE sheets and interpreted the *in vivo* experiments; S.K. supervised all the experimental results and edited the manuscript.

## Supplementary Material

Supplementary Information Supplementary information for Pigment Epithelium-Derived Factor Secreted from Retinal Pigment Epithelium Facilitates Apoptotic Cell Death of iPSC
