# Supplementary material for: Pigment Epithelium-Derived Factor Secreted from Retinal Pigment Epithelium Facilitates Apoptotic Cell Death of iPSC
Source: Sci Rep. 2013 Aug 1;3:2334. doi: 10.1038/srep02334 (PMC3730169; doi:10.1038/srep02334)
Supplement: Supplementary Information — for Pigment Epithelium-Derived Factor Secreted from Retinal Pigment Epithelium Facilitates Apoptotic Cell Death of iPSC [file srep02334-s1.pdf]

Supplementary Information

Pigment Epithelium Derived Factor secreted from Retinal Pigment Epithelium facilitates apoptotic cell death of iPSC

Hoshimi Kanemura, Masahiro J Go, Naoki Nishishita, Noriko Sakai, Hiroyuki Kamao, Yoji Sato, Masayo Takahashi & Shin Kawamata

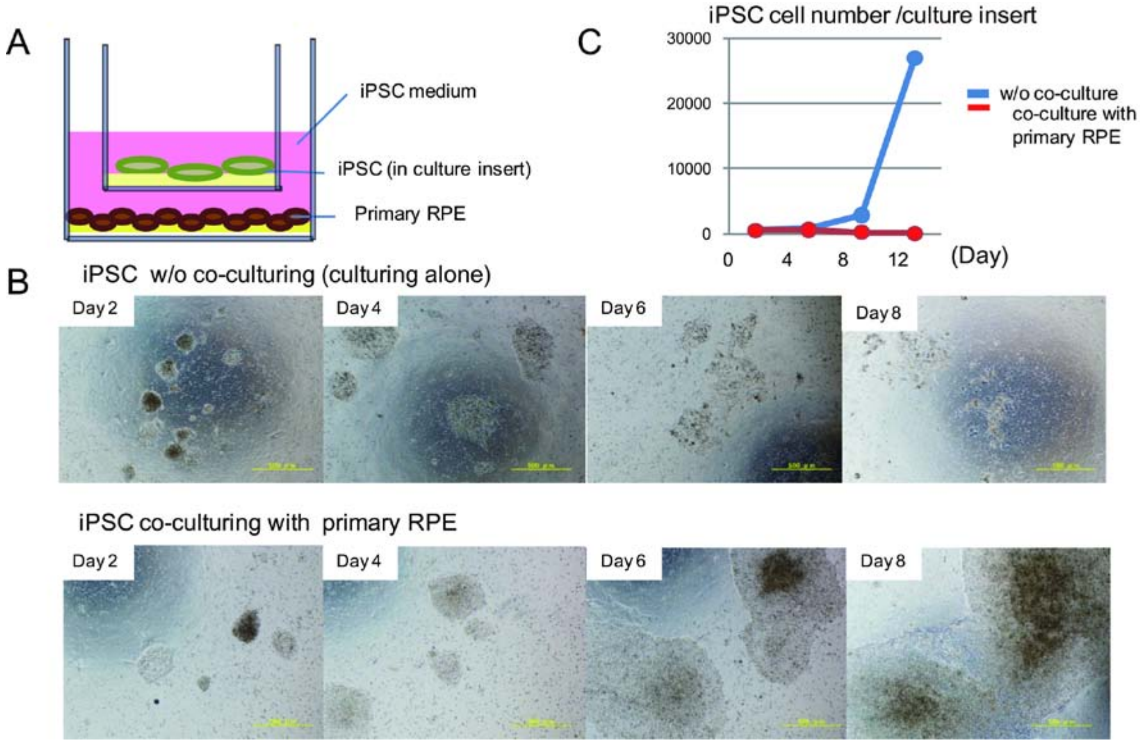

S1. Cell growth of iPSCs co-cultured with primary RPE was perturbed.
(A) Schema for co-culturing iPSC with primary RPE. iPSCs were maintained in culture inserts coated with Matrigel and co-cultured with primary RPE seeded on the bottom of the dishes in iPSC culture medium. (B) Phase-contrast images of iPSC clone 253G1 either cultured alone or co-cultured with primary RPE at designated day of culture. Scale bar = 500 μm. (C) Growth curve of iPSC clone 253G1 co-cultured with primary RPE. Number of iPSC clone 253G1 cells at designated day of culture was scored.

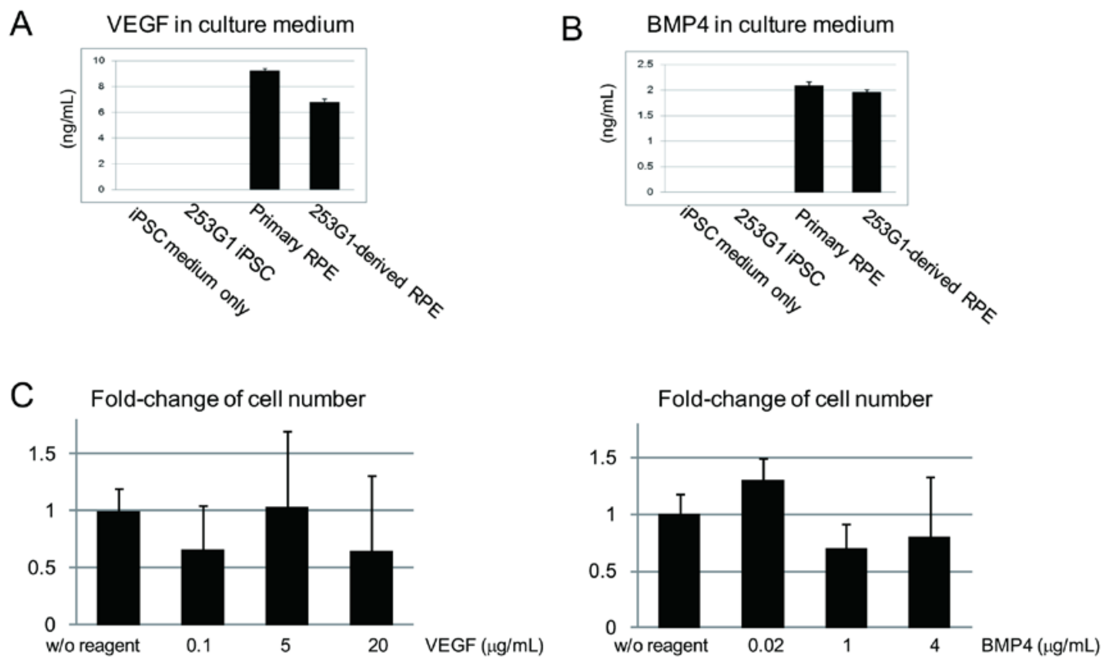

S2. Addition of rVEGF or rBMP4 failed to alter iPSC growth markedly.
VEGF (A) or BMP4 (B) proteins in conditioned media of primary RPE or 253G1-derived RPE cultures after 24 h was measured by ELISA. iPSC medium was used as a negative control. Mean and (SD) from three independent experiments. (C) Fold-change in the number of 253G1 cells cultured either with VEGF (0.1 μg/mL, 5 μg/mL, or 20 μg/mL) or BMP4 (0.02 μg/mL, 1 μg/mL, 4 μg/mL) after six days of culture. Mean results with (SD) from three independent experiments.

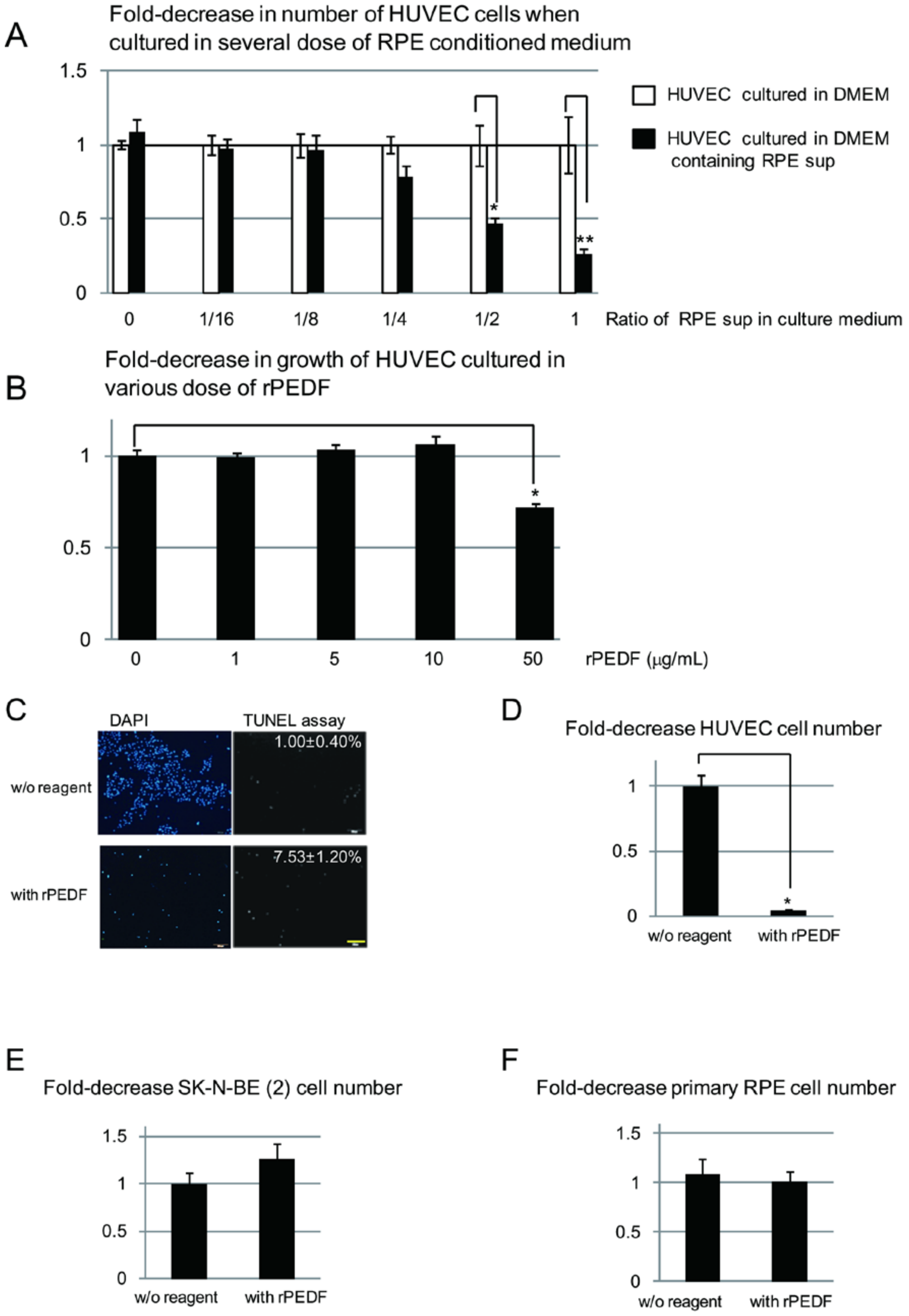
A
Fold-decrease in number of HUVEC cells when cultured in several dose of RPE conditioned medium
1.5
1
0.5
0
HUVEC cultured in DMEM
HUVEC cultured in DMEM containing RPE sup
*
**
0
1/16
1/8
1/4
1/2
1
Ratio of RPE sup in culture medium
B
Fold-decrease in growth of HUVEC cultured in various dose of rPEDF
1
0.5
0
*
0
1
5
10
50
rPEDF (μg/mL)
C
DAPI
TUNEL assay
1.00±0.40%
w/o reagent
7.53±1.20%
with rPEDF
D
Fold-decrease HUVEC cell number
1
0.5
0
*
w/o reagent
with rPEDF
E
Fold-decrease SK-N-BE (2) cell number
1.5
1
0.5
0
w/o reagent
with rPEDF
F
Fold-decrease primary RPE cell number
1.5
1
0.5
0
w/o reagent
with rPEDF

S3. Fifty μg/mL rPEDF possessed a biological effect on HUVEC, not SK-N-BE(2) or primary RPE.

(A) Fold-decrease in the number of HUVECs cultured in M-200 supplemented with LSGS mixed with indicated doses of day two conditioned medium from 253G1-derived RPE cells. Mean result with (SD) of three independent experiments. (B) Fold-decrease in the number of HUVECs cultured in various doses of rPEDF (1, 5, 10, 50 μg/mL) after two days of incubation. Mean results of three independent experiments with (SD). *; $P < 0.05$, **; $P < 0.005$ compared as indicated. (C) Apoptotic death of HUVECs after four days of culture in the presence of rPEDF (50 μg/mL) was examined by TUNEL assay and visualized as white spots. Representative trial of four independent experiments. Scale bar = 200 μm. (D) Fold-decrease in the number of HUVECs cultured with 50 μg/mL of rPEDF after four days of incubation. Mean results with (SD) from three independent experiments. *, $P < 0.005$ compared as indicated. (E) Phase contrast images of neuroblastoma SK-N-BE (2) cultured without or with 50 μg/mL of rPEDF for six days. Fold-decrease in number of neuroblastoma cells in the presence or absence of 50 μg/mL of rPEDF after six days of culture. Mean results with (SD) from three independent experiments. Scale bar = 200 μm. (F) Phase contrast images of primary RPE cultured without or with 50 μg/mL of rPEDF for six days. Fold-decrease in the number of neuroblastoma cells cultured in the presence or absence of 50 μg/mL of rPEDF after six days of incubation. Mean results with (SD) from three independent experiments. Scale bar = 200 μm.

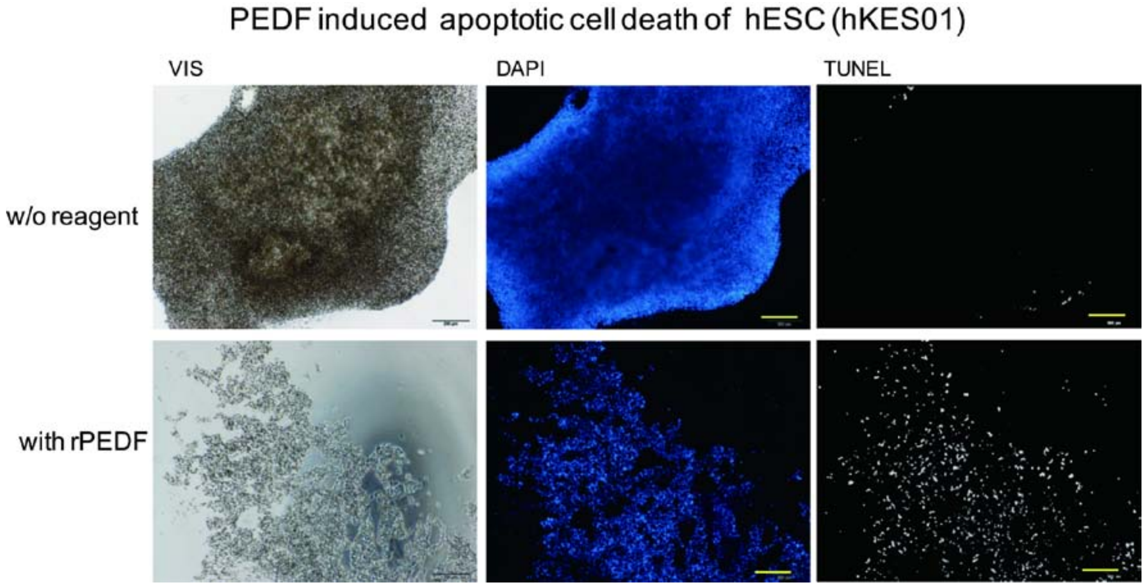

S4. PEDF induced apoptotic cell death of hESC (hKES01).

Apoptotic death of hKES01 cultured without or with 50 μg/mL of rPEDF after four days of incubation was examined by TUNEL assay and visualized as a white spots. Phase contrast images (left panels), DAPI staining (middle panels) and TUNEL assay (right panels). Scale bar = 200 μm.

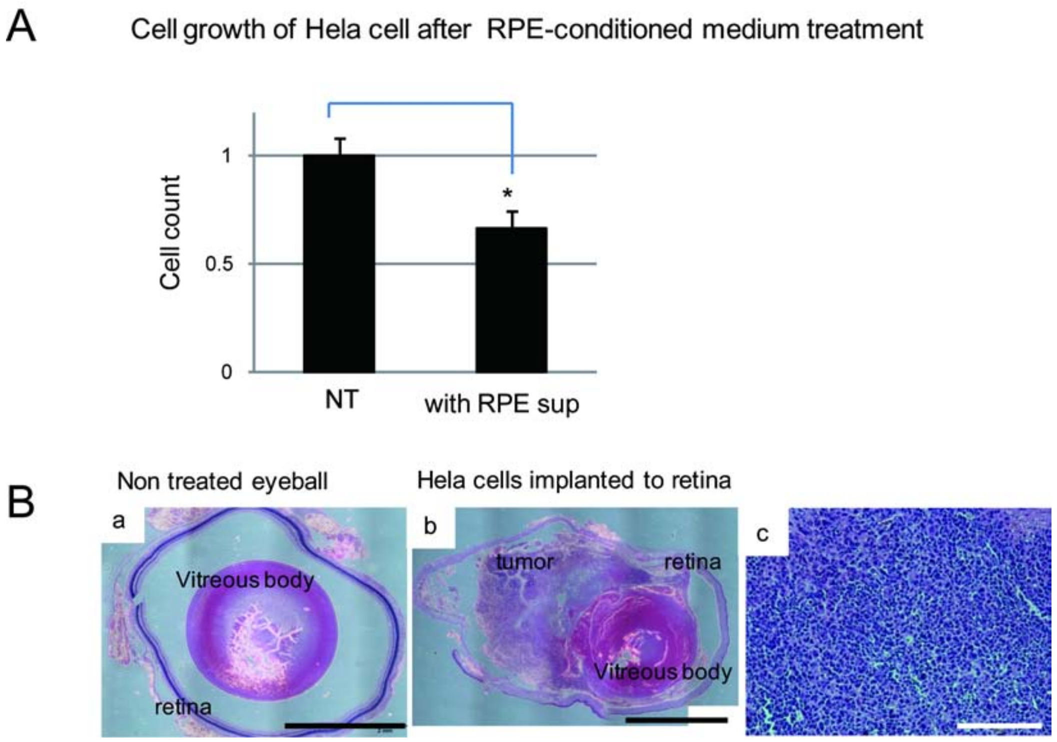

S5. Reduction of HeLa cells number was not drastic compared with that of iPSC under existence of RPE.

(A) Cell growth of HeLa cell after RPE conditioned medium treatment. HeLa cells were treated with or without RPE-conditioned medium for 4 days. Cells number after treatment (with RPE sup) was scored and fold decrease of cell number was shown in a bar graph with an error bar (SD). NT: non-treated HeLa cells. n=3. *; $P < 0.05$ compared as indicated. (B) As few as hundred HeLa cells formed tumor when implanted in retina of nude rat. a) Cross section of non-treated (normal) eyeball. Scale bar = 2 mm. b) Cross section of eyeball injected 100 HeLa cells into retina. 25 weeks after implant. Scale bar = 2 mm. c) Histology of tumor after HeLa cells injection. Scale bar = 200 μm. TPD_{50} for iPSCs is 31623 (n=20, difference between two groups; $P < 0.05$) and TPD_{50} for Hela cells is 32 (n=28 difference between two groups; $P < 0.05$).

Supplemental Table 1

Key soluble molecules identified from gene chip analyses of iPSC clone 253G1, 253G1-derived RPE, and primary RPE are shown.

Development		Primary RPE	253G1-RPE	253G1	hES03
PEDF	pigment epithelium derived factor	8,457	22,448	709	712
BMP2	bone morphogenetic protein 2	1,040	2,426	357	91
VEGFB	vascular endothelial growth factor B	298	498	185	98
MGST2	microsomal glutathione S-transferase 2	1,065	4,644	585	742
GSTM3	glutathione S-transferase mu 3 (brain)	4,002	1,996	121	233

Protease/protease inhibitor		Primary RPE	253G1-RPE	253G1	hES03
EFEMP1	EGF-containing fibulin-like extracellular matrix protein 1	4,237	2,123	0.7	244
PLOD2	procollagen-lysine, 2-oxoglutarate 5-dioxygenase 2	6,311	2,864	696	837
MMP14	matrix metallopeptidase 14 (membrane-inserted)	1,934	995	159	147
CPA4	Carboxypeptidase A4	1,123	1,886	17	73

Cell adhesion/connective tissue protein		Primary RPE	253G1-RPE	253G1	hES03
COL3A1	collagen, type III, alpha 1	5,104	3,871	34	945
SEMA3C	sema domain, immunoglobulin domain (Ig), short basic domain, secreted, (semaphorin) 3C	7,425	4,926	31	79

Complement regulator/immune response		Primary RPE	253G1-RPE	253G1	hES03
CFI	complement factor I	2,556	5,146	0.4	45
APPBP2	amyloid beta precursor protein (cytoplasmic tail) binding protein 2	2,147	2,218	302	500
